# Association Between the Dietary Inflammatory Index and Depression in Mid-Pregnancy: Mediating Effect of Sleep Quality

**DOI:** 10.3390/nu17091434

**Published:** 2025-04-24

**Authors:** Zhitan Zhang, Cong Huang, Hua Zhang, Xun Huang, Zixin Zhong, Fan Xia, Junwei He, Yuxin Ma, Chang Liu, Hongzhuan Tan, Jing Deng, Mengshi Chen, Xianyang Lei

**Affiliations:** 1 Department of Epidemiology and Health Statistics, Xiangya School of Public Health, Central South University, No. 172 Tongzipo Road, Yuelu District, Changsha 410013, China; 18661893668@163.com (Z.Z.); huangcc2000@163.com (C.H.); huangxunxun66@163.com (X.H.); 13574652893@163.com (Z.Z.); 226901005@csu.edu.cn (F.X.); hjw001007@163.com (J.H.); 226911023@csu.edu.cn (Y.M.); 32230094@csu.edu.cn (C.L.); tanhz@mail.csu.edu.cn (H.T.); jingdeng@csu.edu.cn (J.D.); mancechen@foxmail.com (M.C.); 2 Qingdao Huangdao District Market Supervision Administration, No. 627 Binhai Avenue, Qingdao West Coast New District, Qingdao 266427, China; xjdzhh@163.com; 3 Hunan Provincial Key Laboratory of Clinical Epidemiology, Xiangya School of Public Health, Central South University, No. 172 Tongzipo Road, Yuelu District, Changsha 410013, China; 4 Department of Social Medicine and Health Management, Xiangya School of Public Health, Central South University, No. 172 Tongzipo Road, Yuelu District, Changsha 410013, China

**Keywords:** depression, diet/adverse effects, inflammation, pregnancy, sleep quality

## Abstract

Objective: The aim of this study was to assess the association between the Dietary Inflammatory Index (DII) and depression in mid-pregnancy and the mediating effect of sleep quality according to the Edinburgh Postnatal Depression Scale (EPDS). Methods: This was a cross-sectional study conducted in 2017–2019 at a maternal and child health centre in Hunan Province. After applying the inclusion and exclusion criteria, 749 pregnant women were finally included in this study. A multifactorial logistic regression model was used to estimate the odds ratio (OR) and 95% confidence interval (95% CI) of the mid-pregnancy DII on mid-pregnancy depression. Restricted cubic spline plot regression was used to analyse the nonlinearity of the association between DII and depression. Mediation effects models were used to analyse the mediating role of sleep quality. Results: The average age of the 749 pregnant women was 29.42 ± 4.22 years. The mean mid-pregnancy DII was 0.21 ± 1.48 and the overall presenting rate of mid-pregnancy depression was 9.35%. In the final model adjusted for covariates such as maternal age, race, mid-pregnancy body mass index (BMI), occupation, literacy, and mean monthly income, the risk of depression in mid-pregnancy DII Q3 was 3.51 times higher than the mid-pregnancy DII Q4 [OR = 3.51, 95% CI = (1.45 to 8.53)]. A high DII in mid-pregnancy was a risk factor for depression in mid-pregnancy (*p* < 0.05). Restricted cubic spline plot regression analyses showed that the association between mid-pregnancy DII and depression could not be considered nonlinear. Sleep quality may play a mediating role between DII and depression in pregnant women in mid-pregnancy (25.26% of the total effect). Conclusions: A high DII is a risk factor for mid-pregnancy depression. The Dietary Inflammatory Index can influence depression not only directly but also indirectly through the mediating effect of sleep quality on mid-pregnancy depression.

## 1. Introduction

Maternal mental health is an important public health hot topic. According to the WHO, nearly one in five women experience mental health problems during pregnancy [1], with depression being the most common. A meta-analysis by Gavin et al. [2] showed that approximately 13% of women had depressive symptoms during pregnancy or the first year postpartum. Depression during pregnancy is one of the risk factors for postpartum depression, and the resulting incidence of postpartum depression is high [3]. The global pooled prevalence of perinatal depression is 24.7 per cent, and in high- and middle-income countries, the pooled prevalence is also 24.7 per cent [4]. It was found that about 70% of pregnant women who were first screened as depressed by the Edinburgh Postnatal Depression Scale (EPDS) remained depressed until 3 months postpartum. The findings of Xu Jihong et al. [5] in 2019 showed that the detection rate of mid-pregnancy depression was 23.70%. In screening for the prevalence of antenatal depression using validated screening tools and a structured interview, Bennett found that the prevalence of depression was 7.4% in early pregnancy, 12% in mid-pregnancy, and 12.8% in late pregnancy [6]. Differences in this may be related to different study countries and regions, time of year, and screening tools. Mid-pregnancy depression can not only cause pregnancy complications such as gestational diabetes and pre-eclampsia [7] but can also affect foetal growth and development, resulting in adverse pregnancy outcomes such as preterm delivery [8]. Pregnant women with depressive symptoms in mid-pregnancy had 1.72 times the risk of developing gestational diabetes than asymptomatic pregnant women (OR = 1.72, 95% CI: 0.90–3.29) [9].

Risk factors for mid-pregnancy depression include age, education, economic status, family relationships [10], social support [11], and marital status. Diet is an important regulator of chronic inflammation in the organism. Biologically active food components affect the process of inflammatory reactions in the organism, and irrational dietary components (trans fats, cholesterol, saturated fatty acids, etc.) may induce inflammatory reactions involved in the intrinsic mechanisms of depression [12]. A systematic review by Tolkien et al. [13] found that people on a pro-inflammatory diet were 1.4 times more likely to develop depression or exhibit depressive symptoms compared to those on an anti-inflammatory diet. A cohort study of middle-aged women in Australia [14] found that women on an anti-inflammatory diet had an approximately 20 per cent lower risk of depression than women on a pro-inflammatory diet. The Dietary Inflammatory Index (DII) is an evaluation tool to assess the inflammatory potential of an individual’s diet. It classifies foods into 45 dietary components that affect inflammation in the body to quantitatively assess dietary inflammatory potential in relation to disease. Lower scores on the Dietary Inflammatory Index indicate a stronger anti-inflammatory effect; higher scores indicate a stronger pro-inflammatory effect of the diet. Existing studies have found the DII to be associated with depression. A meta-analysis showed that elevated levels of dietary inflammation were associated with a 31% increased risk of depression and that pro-inflammatory diets were more strongly associated with depression in women than in men [15].

The microbial gut–brain axis is a bidirectional neurohumoral communication system that integrates the gastrointestinal tract with the brain and plays a key role in neuropsychiatry [16]. And diet is the primary factor in regulating this axis. A pro-inflammatory diet affects the gut microbiota, influencing neuronal function and synaptic plasticity, which in turn increases the risk of developing depression [17]. Pro-inflammatory diets also activate the innate immune system, triggering low-grade inflammation with chronic non-communicable diseases [18]. Activated immune cells produce too many reactive oxygen species, causing oxidative stress, which affects neuronal cells in the brain and induces depressive symptoms. Inflammatory factors and depression are closely linked. Inflammatory factors are capable of affecting brain function through possible mechanisms such as altering neurotransmitter metabolism and neuropeptide concentrations, over-activation of microglia and astrocytes, changes in monoamine neurotransmitters, and influencing tryptophan metabolism, which in turn induce depressive symptoms [19]. Studies have shown an association between pro-inflammatory cytokine levels in the blood of pregnant women and depressive symptoms [20,21]. Studies have also shown elevated levels of inflammatory factors such as TNFα, IL-6, IL-13, IL-18, IL-12, IL-1RA, and sTNFR2 in depressed patients [22]. The inflammatory cytokines IL-6, QUIN, TNF, and KYN have a high individual accuracy in predicting the future risk and severity of depression in pregnant women, with the use of mid-pregnancy biomarkers to predict symptomatic performance in late pregnancy being particularly prominent [23].

In addition, studies have shown that perinatal depression is associated with the quality of sleep during pregnancy. The risk of prenatal depression is more than three times higher in pregnant women with poor sleep quality compared to those with good sleep quality during pregnancy [24]. Sleep quality can be hypothesised to affect depression by interrupting rapid eye movement sleep (REMS) [25], modulating specific genes [26], and interrupting circadian rhythms [27]. However, evidence on the effects of sleep quality on perinatal depression and its mechanisms remains limited and requires continued research.

In conclusion, the association between mid-pregnancy dietary inflammatory indices and depression is not yet fully clarified. Sleep quality may mediate the association between the Dietary Inflammatory Index and depression in mid-pregnancy. Much of the existing literature examining the relationship between the Dietary Inflammatory Index and depression is based on studies conducted on the public HENS database. We are more interested in discovering what happens in other populations that differ from the NHANS study population in terms of dietary culture, genetics, and environmental factors. Studies exploring the association between the Dietary Inflammatory Index and depression in pregnant women are very limited and most of them have examined postpartum depression. This study combined mid-pregnancy nutritional supplements and dietary nutrients to calculate the mid-pregnancy DII and analyse its association with mid-pregnancy depression in order to find potentially manageable interventions (dietary modifications, sleep hygiene strategies) to improve mid-pregnancy depression.

## 2. Materials and Methods

### 2.1. Ethics Certification

This study was approved by the Medical Ethics Committee of Hunan Maternal and Child Health Hospital, China (No. EC201624). Informed consent was signed with the consent of the study subjects.

### 2.2. Study Design

This was a cross-sectional study. The site of this study was a maternal and child health centre in Hunan Province.

### 2.3. Research Target

Incorporation conditions: (1) Pregnant women who had regular obstetric check-ups at our hospital and delivered at our hospital in 2017–2019; (2) pregnant women aged 18–45 years in the mid-pregnancy stage (24–28 weeks of pregnancy); and (3) normal understanding and expression, signed the informed consent form, and were willing to participate in this study.

Exclusionary conditions: (1) Presence of serious complications of pregnancy or serious physical illness; (2) missing dietary data from the mid-pregnancy questionnaire; and (3) failure to complete the Edinburgh Postnatal Depression Scale.

Sample size: Based on the cross-sectional study sample size formula.

$$n={\left(\frac{Z_{1-\alpha /2}}{\delta }\right)}^{2}\times p\times (1-p)$$

The prevalence of mid-pregnancy depression was 27.30% according to a relevant cross-sectional study [5], so p = 0.273. It was assumed that the permissible error was equal to 5%, i.e., α = 0.05. The required sample size was calculated to be 305 (*n* = 305). Considering a 20% non-response rate, 366 pregnant women were ultimately required. The sample size for this study was 749, which is consistent with the sample size estimate.

### 2.4. Data Collection Questionnaire

The mid-pregnancy questionnaire included a general survey, a survey of influencing factors, and depression status. All data were collected by researchers who received uniform training. After completing the questionnaire, the researcher checked for any omissions or logical errors.

(1) General survey: Demographic characteristics of pregnant women (age, ethnicity, education, occupation, number of pregnancies, number of births, average monthly income);

(2) Surveys and physical examination questionnaires: History of smoking, presence of passive smoking, alcohol consumption before pregnancy, alcohol consumption since pregnancy, sex life, Pittsburgh Sleep Quality Index (PSQI) score, moderate physical housework, gestational hypertension, diagnosis of gestational diabetes, systolic blood pressure, diastolic blood pressure, haemoglobin, serum total cholesterol, low-density cholesterol, high-density cholesterol, triglycerides, CNS-specific protein, glycated haemoglobin;

(3) Food Frequency Questionnaire (FFQ): The FFQ is currently the most commonly used dietary survey method [28] and has been shown to have good reliability and validity for evaluating the dietary and nutrient intake status of pregnant women [29]. In this study, the FFQ was used to collect the dietary intake of the study participants in the past month. In this study, the types of food covered by the Food Frequency Questionnaire (FFQ) were classified into nine categories, specifically covering cereals, potatoes, vegetables, fruits, livestock and meat, seafood, freshwater products, eggs, milk and milk products, soya bean and its products, nuts, oils and fats, salt, water, and beverages. Among them, vegetables and fruits are known anti-inflammatory sources. Conversely, meat is a known pro-inflammatory source. For the frequency option, the expressions ‘never’, ‘once or more per month’, ‘once or more per week’, and ‘once or more per day’ were used. The quantity of each serving was filled in by the study participants themselves. On this basis, we compiled nutritional composition tables of the various nutritional supplements consumed by the study participants through doctors or online shopping. After standardising the nutrient units on the lists, we calculated the nutrient intake of pregnant women in mid-pregnancy through the consumption of nutritional supplements by combining the questionnaires completed by each pregnant woman. Finally, this information was combined with dietary nutrients to calculate the DII, which could reflect the dietary habits, dietary intake, and nutrient data of the respondents in mid-pregnancy more realistically;

(4) Edinburgh Postnatal Depression Scale (EPDS) in mid-pregnancy: Temporarily applicable prenatal depression screening is mainly performed using the Edinburgh Postnatal Depression Scale (EPDS) and the Patient Health Questionnaire (PHQ-9). The Edinburgh Postnatal Depression Scale (EPDS) was developed by Cox et al. [30] and has since been reworked and refined. The revised scale consists of 10 items, each of which is rated on a 4-point scale according to the severity of the symptoms and assigned a score of 0 to 3 (1 and 2 items are reverse scored), and the sum of the scores of the ten items is the total score. The total score of the scale ranges from 0 to 30, with higher scores representing higher levels of depression. The scale has the advantages of simple scoring, being less time-consuming, having no particular time specified for assessment, and having higher specificity [31]. The scale has been confirmed to have high reliability in screening for prenatal depression, with an internal consistency coefficient of 0.76, a content validity of 0.93 [32], and a Cronbach’s alpha coefficient of 0.87. Both national and international studies [31,33,34,35] have concluded that the EPDS excels in perinatal mental health screening and is an effective tool for the initial screening of pregnant women for depressive symptoms during pregnancy and postpartum women;

(5) Pittsburgh Sleep Quality Index (PSQI): This self-assessment scale of sleep quality was developed by Buysse et al. (1989) [36]. The scale consists of 19 items in seven subscales: subjective sleep quality, sleep latency, sleep duration, habitual sleep efficiency, sleep disorders, sleep medication use, and daytime dysfunction. PSQI scores range from 0 to 21, with scores >5 indicating a poor sleep quality [36,37]. The PSQI scale has a good construct validity and reliability in assessing sleep quality in pregnant women [38].

### 2.5. Calculation of the Dietary Inflammatory Index

The procedure for calculating the Dietary Inflammatory Index (DII) for individuals in this study followed the DII calculation procedure in Shivappa et al. [39]. Z-scores for each nutrient component were calculated for the study participants based on their dietary and supplement intake data, compared with the global mean intake and standard deviation of the 45 dietary components’ intake according to a database from populations in 11 countries and regions [40]. That is, Z = (daily intake of a dietary ingredient or nutrient - global average daily per-capita intake of that dietary ingredient or nutrient)/standard deviation of the global average daily per-capita intake of that dietary ingredient or nutrient. The resulting Z-scores were converted to percentiles; to minimise the effect of ‘right skewness’, the resulting percentile values were doubled and then centred by subtracting ‘1’; then, we multiplied by the specific inflammatory effect scores of the corresponding dietary components, and we finally summed to obtain an overall Dietary Inflammatory Index score for all dietary components. Finally, the inflammatory effect scores of all dietary components were summed to obtain the overall Dietary Inflammatory Index score for the individual. Considering the low frequency of consumption of certain food components in the study population and the lack of reliable reference data in the study population, the efficiency of this study was improved while ensuring its scientific validity. In this study, 26 of the 45 food parameters were included in the calculation of the Dietary Inflammatory Index: total fat, saturated fat, monounsaturated fatty acids (MUFAs), polyunsaturated fatty acids (PUFAs), *n*-3 fatty acids, *n*-6 fatty acids, protein, carbohydrates, insoluble dietary fibre, cholesterol, total folate, β-carotene, vitamin B1, vitamin B2, vitamin B6, vitamin B12, vitamin C, vitamin D, vitamin E, niacin, magnesium, iron, zinc, selenium, energy, and vitamin A.

### 2.6. Intermediary and End Variable Definitions

Mid-pregnancy depression: Judgements were made based on the Edinburgh Postnatal Depression Scale score. Scores of ≤12 were classified as non-depressed, whereas Scores of ≥13 were classified as clinically depressed [41].

Sleep quality: Judgements were made based on the Pittsburgh Sleep Quality Index score. Scores of ≤5 were classified as very good sleep quality, 6–10 as good sleep quality, and ≥11 as poor sleep quality [36].

### 2.7. Data Analysis

This study was statistically analysed using SPSS 27.0 and R 4.4.1. Quantitative variables that met normality were described using the mean ± standard deviation; quantitative variables that did not meet normality were described using the median (P25, P75); and categorical variables were described by the frequency (per cent). Continuous variables such as maternal age, BMI, PSQI score, number of pregnancies, and number of deliveries were compared between groups using two independent-samples t-tests or non-parametric tests depending on whether the normality test was satisfied; between-group comparisons were made using chi-square tests for unordered variables such as ethnicity, occupation, literacy, and average monthly income; between-group comparisons were made using non-parametric tests for multicategorical ordinal variables such as overall sleep quality and DIIQ1–Q3. The mid-pregnancy Dietary Inflammatory Index (DII) scores were divided into three groups according to quartiles (P25–P75), i.e., DII Q1 (Quartile 1) for minimum to P25 values, DII Q2 (Quartile 2) for P25 to P75, and DII Q3 (Quartile 3) for P75 to maximum values, where DII Q1 (Quartile 1) is designated as the reference group. Logistic regression models were used to determine the superiority ratio (OR) and 95% confidence intervals (CIs) of the mid-pregnancy Dietary Inflammatory Index in relation to mid-pregnancy depression. We analysed the nonlinear association between DII and depression through restricted cubic spline plot regression by R 4.4.1 software. The SPSS 27.0 macro program Process was used to perform the mediated effects model, to analyse the mediated effects of sleep quality. The test level α = 0.05 was set, and *p* < 0.05 was considered statistically significant.

## 3. Results

### 3.1. General Characteristics of Participants

A total of 771 study participants were sampled in this study. Ultimately, 749 study subjects were included in the statistical analysis due to 2 missing data from the partial diet questionnaire, 19 missing data from the Edinburgh Postnatal Depression Scale (EPDS), and 1 duplicate questionnaire. The basic profile of the study population is shown in Table 1. The average age of pregnant women was 29.42 years. The mean mid-pregnancy DII score was 0.21 ± 1.48. The mean mid-pregnancy EPDS score was 7.55 ± 3.56. The prevalence of mid-pregnancy depression in the study population was 9.35 per cent. The differences in mean monthly income, sleep quality, moderate physical housework, and DII scores were statistically significant between the depressed and non-depressed groups (all *p* < 0.05).

### 3.2. Logistic Regression Analysis of Mid-Pregnancy Depression

A multifactorial logistic regression model was formed with whether pregnant women were depressed in mid-pregnancy as the dependent variable and DII Q1-Q3 in mid-pregnancy as the independent variable, adjusting for covariates. The results are shown in Table 2. The results showed that a high DII in mid-pregnancy was a risk factor for mid-pregnancy depression in pregnant women (*p* < 0.05). Without adjusting for covariates, the risk of depression in mid-pregnancy DII Q3 (pro-inflammatory) was 2.70 times higher than the mid-pregnancy DII Q1 (anti-inflammatory) [OR (95% CI) = 2.70 (1.30 to 5.62)]. The prevalence of depression increases by 26 per cent for every 1 unit increase in the DII. After adjusting for maternal age, ethnicity, mid-pregnancy BMI, occupation, and literacy variables, the risk of depression in mid-pregnancy DII Q3 (pro-inflammatory) was 2.90 times higher than that the mid-pregnancy DII Q1 (anti-inflammatory) [OR (95% CI) = 2.90 (1.35 to 6.25)]. The prevalence of depression increases by 30 per cent for every 1-unit increase in the DII. Finally, after continuing to adjust for average monthly maternal income, number of pregnancies, number of deliveries, passive smoking or not, alcohol consumption since pregnancy, current sexual activity, moderate physical activity, and gestational diabetes diagnostic variables, the risk of depression according to the mid-pregnancy DII Q3 (pro-inflammatory) was 3.51 times higher than that of the mid-pregnancy DII Q1 (anti-inflammatory) [OR (95% CI) = 3.51 (1.45 to 8.53)]. For every 1-unit increase in DII, the prevalence of depression increases by 29 per cent.

### 3.3. Restricted Cubic Spline Plots for Mid-Pregnancy DII and Mid-Pregnancy Depression

**Figure 1:**
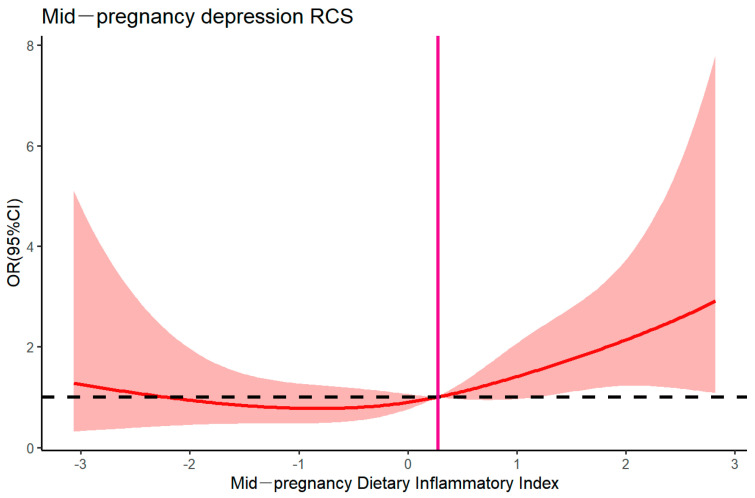
Restricted cubic spline plots for DII and depression.

The mid-pregnancy Dietary Inflammatory Index was used as the horizontal coordinate, and the relative risk of mid-pregnancy depression was used as the vertical coordinate. Age, ethnicity, mid-pregnancy BMI, occupation, literacy level, average monthly income, presence of passive smoking, presence of post-pregnancy alcohol consumption, presence of sexual intercourse, sleep quality (PSQI score division), and presence of moderate physical activity were used as covariates and adjusted in a restricted cubic spline plot. The results showed that overall *p* = 0.045 and nonlinear *p* = 0.271 > 0.05, suggesting that the mid-pregnancy Dietary Inflammatory Index did not have a nonlinear relationship with the risk of mid-pregnancy depression. Based on the results in Figure 1, when DII < 0.277, the risk of mid-pregnancy depression changes slowly with the increase in DII; at DII = 0.277, the cut-off value of the OR ≈ 1; and at DII > 0.277, the risk of mid-pregnancy depression increases significantly with the increase in DII. Therefore, it is appropriate to control the mid-pregnancy DII to be below 0.277.

### 3.4. The Mediating Role of Sleep Quality Between DII and Depression in Mid-Pregnancy

In order to explore the underlying mechanism of the significant positive effect of the DII on depression, sleep quality was further introduced as a mediating variable to be substituted into the structural equation modelling in this study. Adjusting for maternal age, ethnicity, BMI, occupation, literacy, and average monthly income, Model 4 in the SPSS macro program Process was used to test the mediating effect, and the mediating role of sleep quality between the DII and depression in mid-pregnancy was verified and analysed according to the bootstrap method by Hayes.

**Figure 2:**
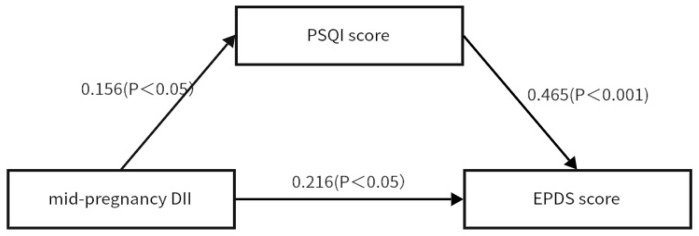
Path coefficient plots for DII, sleep quality, and depression.

The path coefficients for sleep quality between the DII and mid-pregnancy depression variables are shown in Figure 2.

According to Table 3, the upper and lower limits of the bootstrap 95% confidence intervals for the mediating effect of the DII on depression and sleep quality in mid-pregnancy do not contain 0, indicating that the DII not only acts as a direct effector on depression but can also act indirectly on depression through the sleep quality mediating variable. This direct effect (0.216) and mediating effect (0.073) accounted for 74.74% and 25.26% of the total effect (0.289), respectively. The implication is that when the DII is high, this may interfere with sleep regulation mechanisms, leading to a reduced sleep quality. The specific physiological and psychological characteristics of pregnancy make the mediating role of sleep quality between the DII and mid-pregnancy depression more prominent, leading sleep quality to mediate a proportion of the total effect.

## 4. Discussion

The rate of mid-pregnancy depression in this study was 9.35%, which is close to the findings of Gu Shui-Qin et al. [42] in Zhejiang, China (10.44%). This may be due to the consistency of using the same EPDS score ≥ 13 as the basis of division in similar populations. The present study found that pregnant women with mid-pregnancy depression had a significantly higher DII than non-depressed pregnant women. After adjusting for various potential confounders, the risk of depression was 3.51 times higher in the highest mid-pregnancy DII group than in the lowest mid-pregnancy DII group. This is consistent with some of the previous studies reporting an increased risk of depression in people on the most pro-inflammatory diets [43,44]. It provides further evidence that unhealthy dietary patterns, i.e., diets with a higher inflammatory potential, may increase the risk of depression during pregnancy.

Pregnancy is a special time when the immune status is prone to change. Inflammation may play an extremely important role in mediating between diet and mid-pregnancy depression. Pro-inflammatory cytokines produced by immune cells can inhibit the brain’s secretion of neurotransmitters such as norepinephrine and dopamine, ultimately leading to depression [45]. Chronic inflammation induced by poor dietary habits may negatively affect the emotional state of pregnant women and further increase the susceptibility to depression by interfering with neurotransmitter metabolism, disrupting the balance of the neuroendocrine system, and inhibiting neuroplasticity. National and international studies have shown that increased concentrations of pro-inflammatory cytokines play a key role in the pathogenesis of depression [46,47]. A large cohort study [48] noted that the processed food intake was significantly and positively associated with the depression risk. Specifically, for every 10 per cent increase in processed food intake, the risk of depression rose by 21 per cent. Those with a Western-style diet or unhealthy eating patterns typified by high energy, fat, sugar, refined grains, and alcohol are at significantly increased risk of depression [49,50]. Diet is an important modifiable factor in depression, and a rational diet is key to regulating the regression of chronic inflammation in the body [19]. Taking the Mediterranean dietary pattern as an example, which emphasises a high intake of fruits, vegetables, whole grains, fish, and lean meat, people who adhere to the Mediterranean dietary style over a long period of time are relatively less likely to suffer from depression [51,52]. This is a good example of the positive role of healthy dietary patterns in preventing mood disorders such as depression during pregnancy.

The mediating effect of sleep quality in the association between the Dietary Inflammatory Index and depression is an important finding of this study. A high DII diet may lead to a decreased sleep quality by triggering an inflammatory response in the body that interferes with sleep regulation mechanisms, such as affecting melatonin secretion. Women are at a greater risk of sleep disorders than men, and anatomical, physiological, hormonal, and psychological problems associated with pregnancy can exacerbate sleep problems and increase as pregnancy progresses [53,54]. In turn, a reduced sleep quality can further affect the emotional stability of pregnant women and increase the risk of depression. The revelation of this mediating pathway suggests that improving sleep quality may offer a potential target for intervention to mitigate the effects of a high inflammatory diet on maternal depression. Novel intervention therapies such as reading books and performing health and wellness exercises [55] and yoga [56] have also emerged in recent years to improve sleep quality.

It was also found during the course of this study that an average monthly income of <10,000 RMB caused mid-pregnancy depression in pregnant women. The physical discomforts and financial burdens of pregnancy exacerbate worries about work and life, which in turn may trigger depressive symptoms [57]. Also, several studies [58,59,60,61,62] have concluded that pregnant women with a lower socioeconomic status or lower income are at a higher risk of prenatal depression. Social support as an emotional coping mechanism can enhance the quality of life of pregnant women and reduce their pregnancy stress levels [63]. Accordingly, the government and society can provide certain social service support to families of pregnant women. Family members should provide more care, attention, and support to pregnant women so that they can have a good emotional experience.

In recent years, signs of inflammation in the blood have been shown to predict and identify major depression during pregnancy. Qiong Sha et al. [23] found that the combination of cytokines and kynurenine metabolites in mid-pregnancy accurately predicted late-pregnancy depression, with a probability > 99% and a ROC AUC > 0.8. Cytokines and tryptophan metabolites predict depression in pregnancy and can be used as clinical markers of risk. Further longitudinal studies, such as prospective cohort studies, should be conducted in the future to more accurately validate the assessment of causality around pregnant women with depression. In-depth investigation of the underlying inflammatory mechanisms, with interventional studies of the impact of dietary modification or sleep improvement on depression, is aimed at exploring strategies to effectively improve the mental health of pregnant women.

## 5. Limitations

Firstly, as this was a cross-sectional study, we cannot establish causality. Secondly, the reliance on self-reports for the dietary data (FFQ), depression scale (EPDS), and sleep quality (PSQI) may introduce recall bias. The DII score is also based on a global database, which may have limitations in its applicability to our specific group of Chinese pregnant women. Furthermore, James R Hébert et al. [64] pointed out that when the Dietary Inflammatory Index algorithm was changed, this could lead to biased results. Finally, it has been suggested that cooking methods may also alter the inflammatory component of food, and the lack of data on cooking methods may thus present a limitation [65].

## 6. Conclusions

A high mid-pregnancy Dietary Inflammatory Index (DII) is a risk factor for mid-pregnancy depression. The Dietary Inflammatory Index can influence depression not only directly but also indirectly through the mediating effect of sleep quality on mid-pregnancy depression. Pregnant women with a high DII have a high prevalence of mid-pregnancy depression compared to those with a low DII, and the implementation of an anti-inflammatory diet may serve as an intervention strategy for pregnant women to prevent mid-pregnancy depressive episodes.

## Figures and Tables

**Table 1:** General characteristics of subgroups of pregnant women with depression in mid-pregnancy.

Characteristics	Groups		Overall (*n* = 749)	Statistical Value	*p* Value
	Non-Depressed Group (*n* = 679)	Depression Group (*n* = 70)			
Age	29.45 ± 4.23	29.16 ± 4.12	29.42 ± 4.22	0.548	0.584
Ethnicity				0.038	0.846
Han ethnic group	649 (95.58)	66 (94.29)	715 (95.46)		
Other	30 (4.42)	4 (5.71)	34 (4.54)		
BMI	23.40 ± 3.08	23.66 ± 2.80	23.42 ± 3.05	−0.686	0.493
Occupation				3.143	0.370
Cadre or career staff	219 (32.25)	16 (22.86)	235 (31.38)		
Employee/worker/commercial	314 (46.24)	38 (54.29)	352 (47.00)		
Unemployed/housewife	79 (11.63)	10 (14.29)	89 (11.88)		
Other	67 (9.87)	6 (8.57)	73 (9.75)		
Education level				0.330	0.848
High school and below	88 (12.96)	9 (12.86)	97 (12.95)		
College or university	518 (76.29)	55 (78.57)	573 (76.50)		
Master’s/PhD students	73 (10.75)	6 (8.57)	79 (10.55)		
Average monthly income				12.840	<0.001 ***
<10,000 RMB	383 (56.41)	55 (78.57)	438 (58.48)		
≥10,000 RMB	296 (43.59)	15 (21.43)	311 (41.52)		
PSQI score	5.88 ± 2.65	8.17 ± 3.10	6.09 ± 2.77	−6.565	<0.001 ***
Sleep quality (PSQI score classification)				−5.713	<0.001 ***
Well	319 (49.38)	12 (18.18)	331 (46.49)		
Preferably	291 (45.05)	39 (59.09)	330 (46.35)		
Mediocre	36 (5.57)	15 (22.73)	51 (7.16)		
Number of pregnancies	2.00 ± 1.17	2.13 ± 1.33	2.01 ± 1.19	−0.863	0.388
Number of times an item is produced	0.39 ± 0.51	0.46 ± 0.56	0.40 ± 0.52	−1.032	0.302
History of smoking				0.154	0.694
no	670 (98.67)	70 (100.00)	740 (98.80)		
yes	9 (1.33)	0 (0.00)	9 (1.20)		
Passive smoking				2.777	0.096
no	570 (84.32)	52 (76.47)	622 (83.60)		
yes	106 (15.68)	16 (23.53)	122 (16.40)		
Drinking alcohol before pregnancy				1.130	0.288
no	657 (97.33)	66 (94.29)	723 (97.05)		
yes	18 (2.67)	4 (5.71)	22 (2.95)		
Drinking alcohol since pregnancy				<0.001	1.000
no	631 (97.23)	65 (97.01)	696 (97.21)		
yes	18 (2.77)	2 (2.99)	20 (2.79)		
Current sex life				2.810	0.094
no	592 (87.19)	56 (80.00)	648 (86.52)		
yes	87 (12.81)	14 (20.00)	101 (13.48)		
Moderate physical housework				4.508	0.034 *
no	386 (56.85)	49 (70.00)	434 (58.02)		
yes	293 (43.15)	21 (30.00)	314 (41.98)		
Moderate physical exercise				0.563	0.453
no	657 (97.91)	67 (95.71)	724 (97.71)		
yes	14 (2.09)	3 (4.29)	17 (2.29)		
Hypertensive pregnancy				1.477	0.224
no	567 (95.94)	59 (100.00)	626 (96.31)		
yes	24 (4.06)	0 (0.00)	24 (3.69)		
Gestational diabetes				0.568	0.451
no	585 (86.16)	58 (82.86)	643 (85.85)		
yes	94 (13.84)	12 (17.14)	106 (14.15)		
Systolic blood pressure	113.26 ± 11.05	110.78 ± 10.10	113.04 ± 10.98	1.770	0.077
Diastolic blood pressure	72.22 ± 9.62	71.30 ± 7.89	72.14 ± 9.48	0.759	0.448
Haemoglobin	116.75 ± 8.08	115.12 ± 7.74	116.61 ± 8.06	1.492	0.136
Anaemic				0.701	0.402
no	526 (83.89)	47 (79.66)	573 (83.53)		
yes	101 (16.11)	12 (20.34)	113 (16.47)		
Total serum cholesterol	5.81 ± 1.00	5.49 ± 1.28	5.78 ± 1.03	1.567	0.118
Low-density cholesterol	3.21 ± 0.87	3.04 ± 0.85	3.20 ± 0.87	1.011	0.313
High-density cholesterol	2.19 ± 0.51	2.26 ± 0.83	2.19 ± 0.55	−0.455	0.653
Triglyceride	2.45 ± 1.43	2.55 ± 0.72	2.46 ± 1.38	−0.352	0.725
CNS-specific protein	0.26 ± 0.21	0.18 ± 0.02	0.25 ± 0.19	0.902	0.372
Glycated haemoglobin	4.90 ± 0.33	4.88 ± 0.35	4.90 ± 0.33	0.403	0.687
DII score	0.16 ± 1.47	0.65 ± 1.51	0.21 ± 1.48	−2.622	0.009 **
DII subgroup				−2.840	0.005 **
DII Q1	176 (25.92)	11 (15.71)	187 (24.97)		
DII Q2	343 (50.52)	32 (45.71)	375 (50.07)		
DII Q3	160 (23.56)	27 (38.57)	187 (24.97)		
EPDS score	6.86 ± 2.94	14.27 ± 1.30	7.55 ± 3.56	38.680	<0.001 ***

Note: *: *p* < 0.05; **: *p* < 0.01; ***: *p* < 0.001.

**Table 2:** Odds ratio and 95% CI of mid-pregnancy depression according to DII as continuous variable and quartiles.

	Model 1			Model 2			Model 3		
	β	OR (95CI%)	*p*	β	OR (95CI%)	*p*	β	OR (95CI%)	*p*
Continuous of DII	0.23	1.26 (1.06~1.51)	0.009	0.26	1.30 (1.08~1.56)	0.006	0.26	1.29 (1.06~1.58)	0.012
Quartile of DII									
Q1		Ref.			Ref.			Ref.	
Q2	0.40	1.49 (0.74~3.03)	0.268	0.41	1.50 (0.71~3.16)	0.283	0.66	1.94 (0.82~4.61)	0.132
Q3	0.99	2.70 (1.30~5.62)	0.008	1.07	2.90 (1.35~6.25)	0.006	1.26	3.51 (1.45~8.53)	0.005

Note: No variables were adjusted in Model 1; Model 2 was adjusted for the maternal age, ethnicity, mid-pregnancy BMI, occupation, and literacy; Model 3 continues Model 2 and adds adjustments for the average monthly income of pregnant women, number of pregnancies, number of births, passive smoking or not, alcohol consumption since pregnancy, current sex life, moderate physical activity, and gestational diabetes diagnosis.

**Table 3:** Breakdown of total, direct, and mediated effects.

	Efficiency Value	SE	95%CI		Effect Size
			LLCI	ULCI	
Total effect	0.289	0.090	0.113	0.465	
Direct effect	0.216	0.084	0.052	0.381	74.74%
Intermediary effect	0.073	0.034	0.007	0.142	25.26%

## Data Availability

The datasets generated and analysed during the current study are available from the corresponding author upon reasonable request.

## Abbreviations

Abridged	Full name
BMI	Body Mass Index
DII	Dietary Inflammatory Index
EPDS	Edinburgh Postnatal Depression Scale
FFQ	Food Frequency Questionnaire
PSQI	Pittsburgh Sleep Quality Index
PHQ-9	Patient Health Questionnaire-9
